# Molecular and morphological convergence to sulfide-tolerant fishes in a new species of *Jenynsia* (Cyprinodontiformes: Anablepidae), the first extremophile member of the family

**DOI:** 10.1371/journal.pone.0218810

**Published:** 2019-07-10

**Authors:** Gastón Aguilera, Guillermo Enrique Terán, Juan Marcos Mirande, Felipe Alonso, Sina Rometsch, Axel Meyer, Julian Torres-Dowdall

**Affiliations:** 1 Fundación Miguel Lillo - Unidad Ejecutora Lillo (CONICET), San Miguel de Tucumán, Tucumán, Argentina; 2 Instituto de Bio y Geociencias del NOA (IBIGEO-CONICET), Rosario de Lerma, Salta, Argentina; 3 Chair of Zoology and Evolutionary Biology, Department of Biology, University of Konstanz, Konstanz, Germany; 4 Hector Fellow Academy, Karlsruhe, Germany; Southwest University, CHINA

## Abstract

Freshwater sulfide springs have extreme environmental conditions that only few vertebrate species can tolerate. These species often develop a series of morphological and molecular adaptations to cope with the challenges of life under the toxic and hypoxic conditions of sulfide springs. In this paper, we described a new fish species of the genus *Jenynsia*, Anablepidae, from a sulfide spring in Northwestern Argentina, the first in the family known from such extreme environment. *Jenynsia sulfurica* n. sp. is diagnosable by the lack of scales on the pre-pelvic area or the presence of a single row of scales, continuous or not, from the isthmus to the bases of the pelvic fins. Additionally, it presents a series of morphological and molecular characteristics that appear convergent with those seen in other fish species (e.g., Poeciliids) inhabiting sulfide springs. Most notably, *J*. *sulfurica* has an enlarged head and postorbital area compared to other fish of the genus and a prognathous lower jaw with a hypertrophied lip, thought to facilitate respiration at the air-water interface. Analyses of *cox1* sequence showed that *J*. *sulfurica* has two unique mutations resulting in amino acid substitutions convergent to those seen in Poeciliids from sulfide springs and known to provide a physiological mechanism related to living in sulfide environments. A phylogenetic analysis, including molecular and morphological characters, placed *J*. *sulfurica* as sister taxa to *J*. *alternimaculata*, a species found in nearby, non-sulfide habitats directly connected to the sulfide springs. Thus, it can be inferred that the selection imposed by the presence of H_2_S has resulted in the divergence between these two species and has potentially served as a barrier to gene flow.

## Introduction

Extremophile species inhabiting environments with abiotic conditions lethal for most organisms, are of great interest to physiologists, ecologists and evolutionary biologists [1, 2]. These species offer valuable information on the limits of tolerance to abiotic conditions (e.g., [3]), on the process of adaptive divergence (e.g, [4]), and in the end, about the predictability of evolution (e.g., [5]). In fact, species inhabiting extreme ecological conditions have provided some very interesting cases of evolutionary convergence at different levels of biological organization, from molecular changes, to physiology, morphology, and performance [6, 7].

Most extremophiles are prokaryotic organisms [8], however, also a significant number of invertebrates and vertebrates successfully colonized extreme environments (e.g., [9]). Among the later, teleost fishes have invaded a range of extreme environments (see [2]). Freshwater sulfide springs, having high concentrations of hydrogen sulfide, are among such extreme environments that were successfully colonized by only a handful of species [10]. Although hydrogen sulfide is commonly found in low abundance in many habitats, and can be detoxified by most organisms at these lower concentrations [11, 12], elevated concentrations are extremely toxic by affecting cellular respiration [13, 14]. Additionally, sulfide springs often have elevated temperatures and decreased dissolved oxygen levels [15], which magnifies the toxicity of hydrogen sulfide, given that the detoxification process requires its oxidation [13]. Only four fish families have evolved adaptations to cope with these extreme conditions: Cyprinodontidae (four species), Poeciliidae (13 species), Synbranchidae (one species) [10], and Rivulidae (1 species) [16]. Among these, Poeciliidae are the most studied family, and some species evolved a series of adaptations that enable them to efficiently acquire oxygen in the hypoxic conditions of sulfide springs. Most species from sulfide springs have enlarged heads that allow for an increase in gill area to maximize oxygen uptake in these oxygen-poor environments [15, 17, 18]. Associated to this, fish in sulfide springs often present modifications of the mouth (e.g., enlarged lower jaw, hypertrophic lips, mouth appendages) that are thought to facilitate respiration at the water-air interface, where oxygen concentrations are elevated compared to the rest of the water column [18–20]. Moreover, there is convergent molecular evolution among sulfide spring fish species at the cytochrome oxidase complex (*cox*), a primary target of hydrogen sulfide toxicity in the respiratory chain [21]. Convergent amino acid substitutions in the *cox1* protein across different sulfide-adapted *Poecilia* species have been proposed to reduce susceptibility to hydrogen sulfide.

Here, we describe a new fish species from a thermal sulfide spring (having high concentrations of H_2_S) in Northwestern Argentina. This is the first species in the family Anablepidae (Cyprinidontiformes) to be found in this type of habitat; thus, adding a new family to the diversity of fish inhabiting sulfide springs. It is a livebearing fish of the genus *Jenynsia* Günther [22], which currently is composed of 14 valid species, considering the last species described (i.e. *J*. *darwini* Amorim) and the recognition of *J*. *multidentata* (Jenyns) as a junior synonym of *J*. *lineata* (Jenyns) [23]. The genus is distributed in South America from the state of Rio de Janeiro, Brazil, to Río Negro, Argentina, and from coastal Atlantic drainages at sea level to rivers bordering the Andean region, from southern Bolivia to central Argentina [23, 24], encompassing a range of altitudes from 0–3000 m.a.s.l. (pers. obs.).

Despite this wide geographic range of distribution exhibited by members of the genus *Jenynsia*, almost all species have relatively restricted distributions with the exception of the *J*. *lineata* species-complex, which ranges in distribution from fresh to brackish and marine waters [23–26]. Some species of the genus can also be found in environments with a great variation in air temperature. Hued & Bistoni [27] described *J*. *lineata* as an environmentally tolerant species following the criteria of Karr et al. [28], since it can be found in a wide range of water conditions, including even polluted waters. Thus, members of the genus *Jenynsia*, like other cyprinodontiform fishes (e.g., [15, 29–31]), can tolerate environmental conditions that are generally harmful to other fishes. However, no anablepid species was previously known to occur in environments containing high concentrations of hydrogen sulfide. Here, we first describe the newly discovered species of *Jenynsia* fish from a sulfide spring in Northern Argentina; then, we show that the mitochondrial *cox1* gene of the new species has amino acid substitutions that are convergent to those seen in species of Poeciliidae adapted to sulfide springs [10, 15, 17, 18]; and finally we provide a new phylogenetic hypothesis based on the morphological data matrix of Ghedotti [25] and posteriorly modified by Aguilera *et al*. [24], with the addition of available DNA data.

## Results

### *Jenynsia sulfurica*, new species

**Fig 1:**
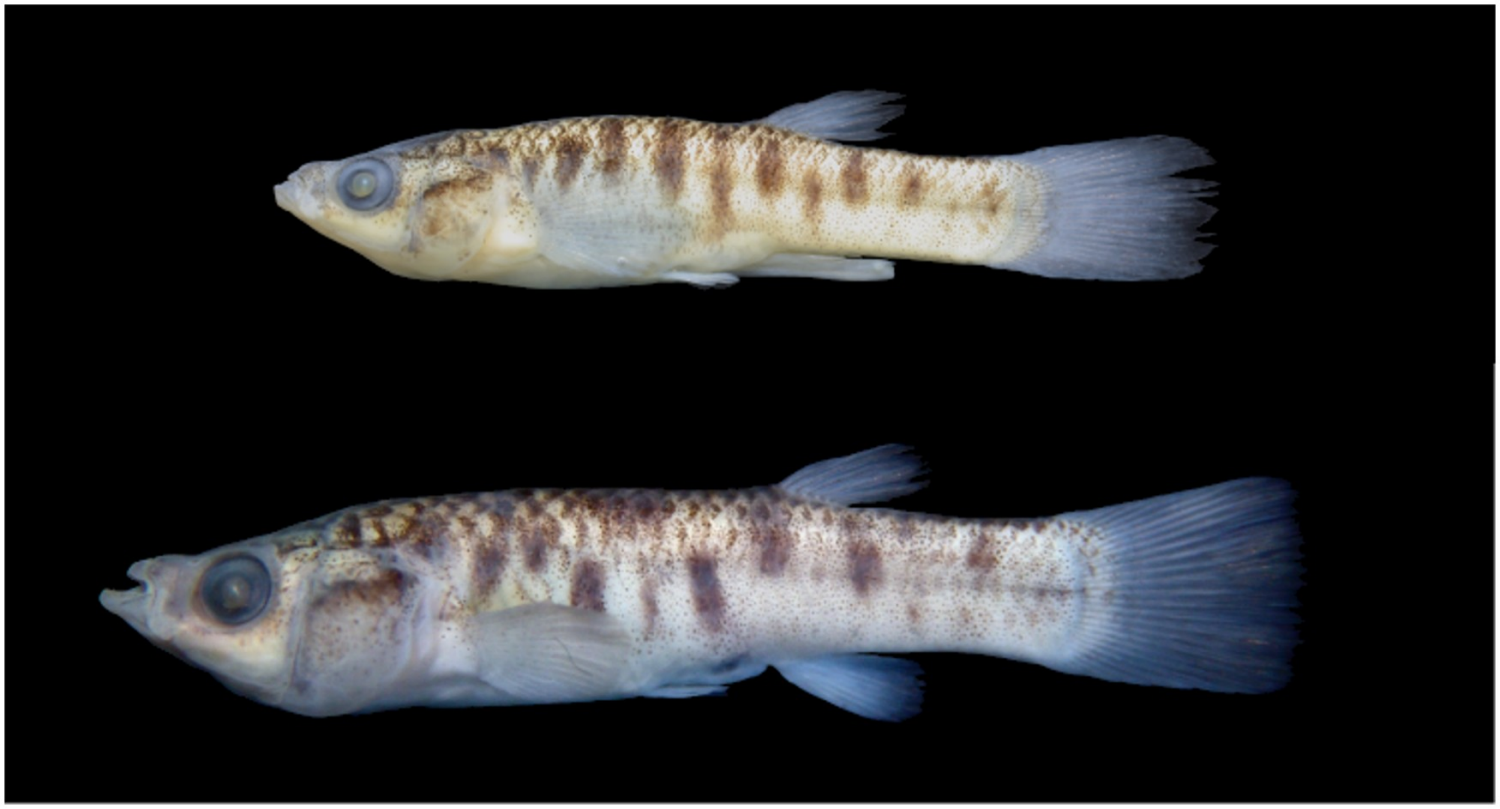
Preserved holotype and paratype specimens of *Jenynsia sulfurica* sp. nov. Above: holotype CI-FML 7286, male, 21.6 mm SL; below: paratype CI-FML: 7287, female, 32.8 mm SL, from the La Quinta lagoon, Santa Barbara department, Jujuy province, Argentina.

**Fig 2:**
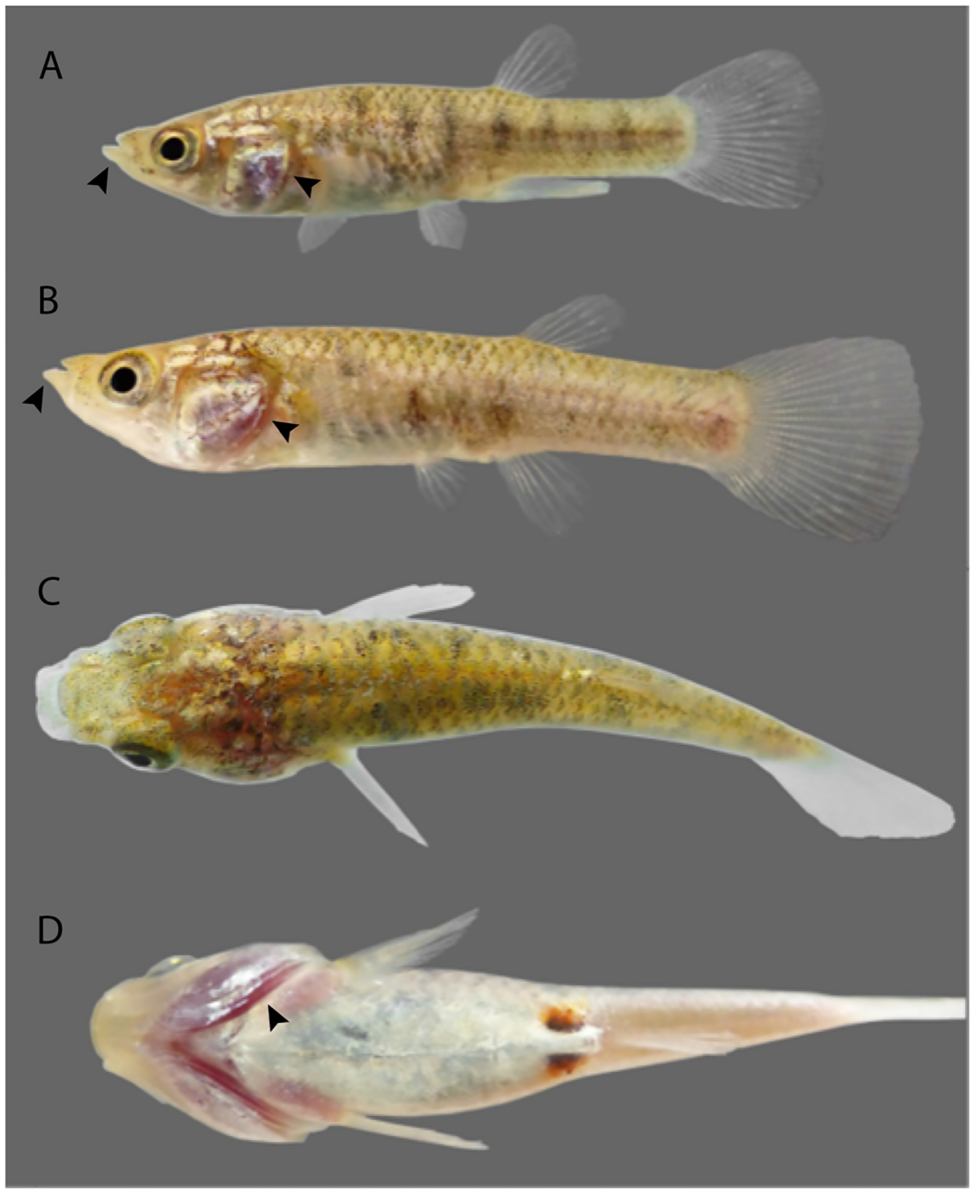
Live specimens of the sulfide-tolerant species *Jenynsia sulfurica* sp. nov. Lateral view of a (A) male and (B) female individual, exhibiting large heads and a prognathous lower jaw with a hypertrophied lip (indicated by arrows) which facilitate respiration at the air-water interface. (C) dorsal and (D) ventral view of a female individual.

urn:lsid:zoobank.org:act: 1F6C69F5-C697-49E8-91F9-E77A502192F8 (Figs 1 and 2).

### Holotype

CI-FML 7286 (Fig 1), 21.6 mm SL, Laguna La Quinta, thermal system at western flank of the Santa Bárbara hills (23°53'3.03"S, 64°28'2.74"W), Santa Bárbara Department of Jujuy Province, Northwestern Argentina. Col: G. Aguilera, J.M. Mirande, G.E. Terán, F. Alonso. November 13, 2016.

### Paratypes

CI-FML 7287 (Fig 1), 19 ex, 20.4–32.8 mm SL, IBIGEO-I 465, 10 ex, 18.7–29.9 mm SL. All collected with holotype.

### Diagnosis

The genus *Jenynsia* is subdivided into two subgenera, one containing five species only known from inland habitats in Brazil (*Plesiojenynsia*) and a more widely distributed subgenus including nine previously described species (*Jenynsia*). The new species herein described (Fig 1) presents the three synapomorphies considered by Ghedotti [25] as diagnostic for the subgenus *Jenynsia*: (1) a modified sixth anal-fin ray segmented on its proximal quarter; (2) unsegmented on its distal quarter in adult males and (3) the vertically inclined proximal radials associated with the first six anal-fin rays in the gonopodium. Also, in the analyses under both, equal and implied weightings, the additional synapomorphies proposed by Aguilera *et al*. [24] for the subgenus *Jenynsia* were recovered (i.e. character state 17–1; 47–1; 51–1 and 64–2; S1 Table). In the new species herein described, character 64 is reversed to state 0 (S1 Table).

**Fig 3:**
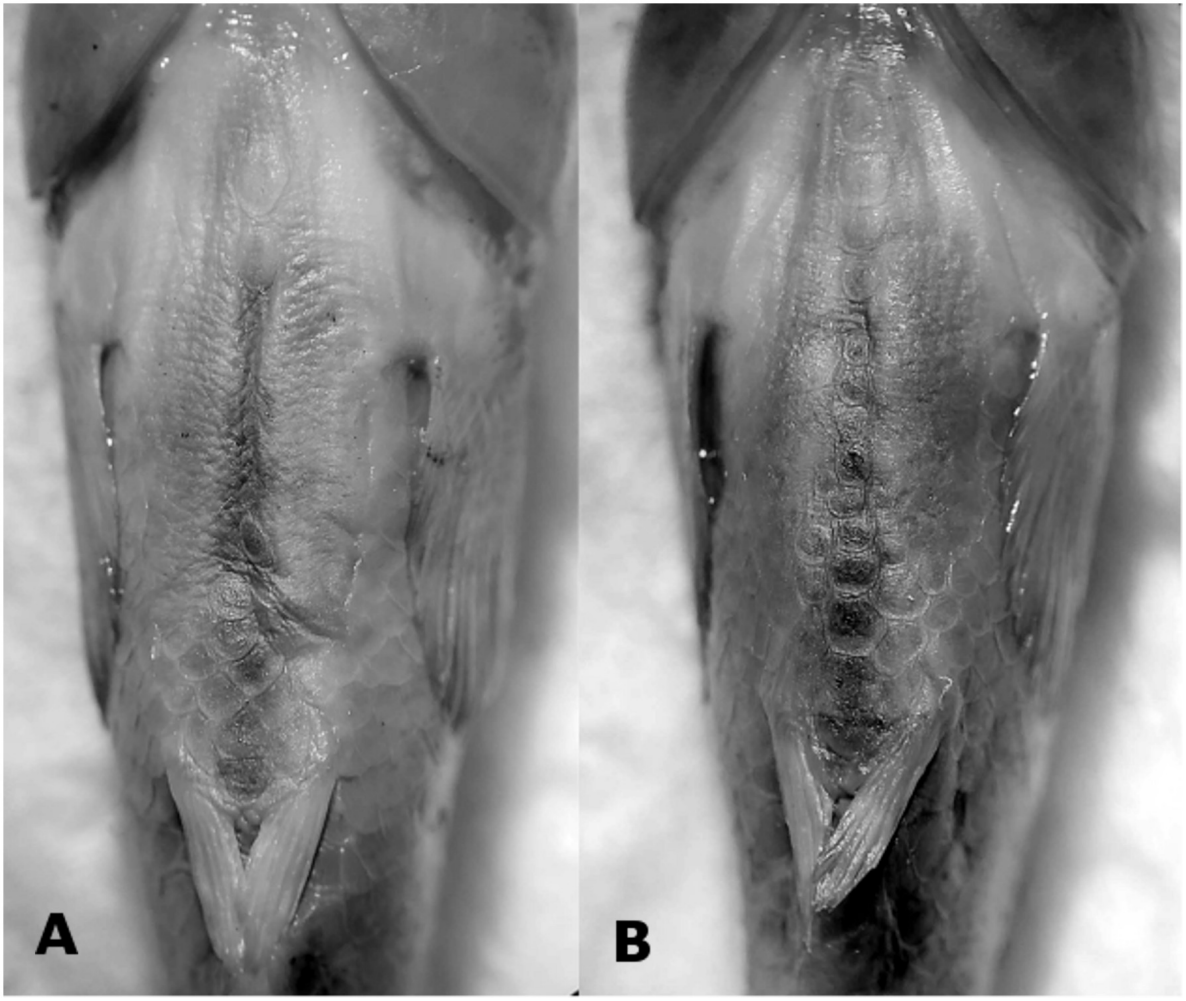
*Jenynsia sulfurica* can be diagnosed by the (almost) complete absence of scales in the abdominal area. Ventral surface photograph of (A) IBIGEO-I 465, female, 25.4 mm; and (B) CI-FML 7287, female, 24.0 mm SL, from the La Quinta lagoon, Santa Barbara department, Jujuy province, Argentina.

The new species is diagnosable from all other species of the genus by the absence of scales on the ventral surface of the body or the presence of a single row of scales, continuous or not, from the isthmus to the pelvic-fin bases (vs. completely scaled ventral surface of body in all species of *Jenynsia*; Fig 3). Additionally, *J*. *sulfurica* presents a unique coloration pattern, exhibiting eight to eleven irregular blotches along the mid-lateral surface of the body. These are formed by dark-brown chromatophores, ranging from rounded spots to vertical bars spreading up to three scales in depth (vs. different configuration pattern; Fig 1).

**Fig 4:**
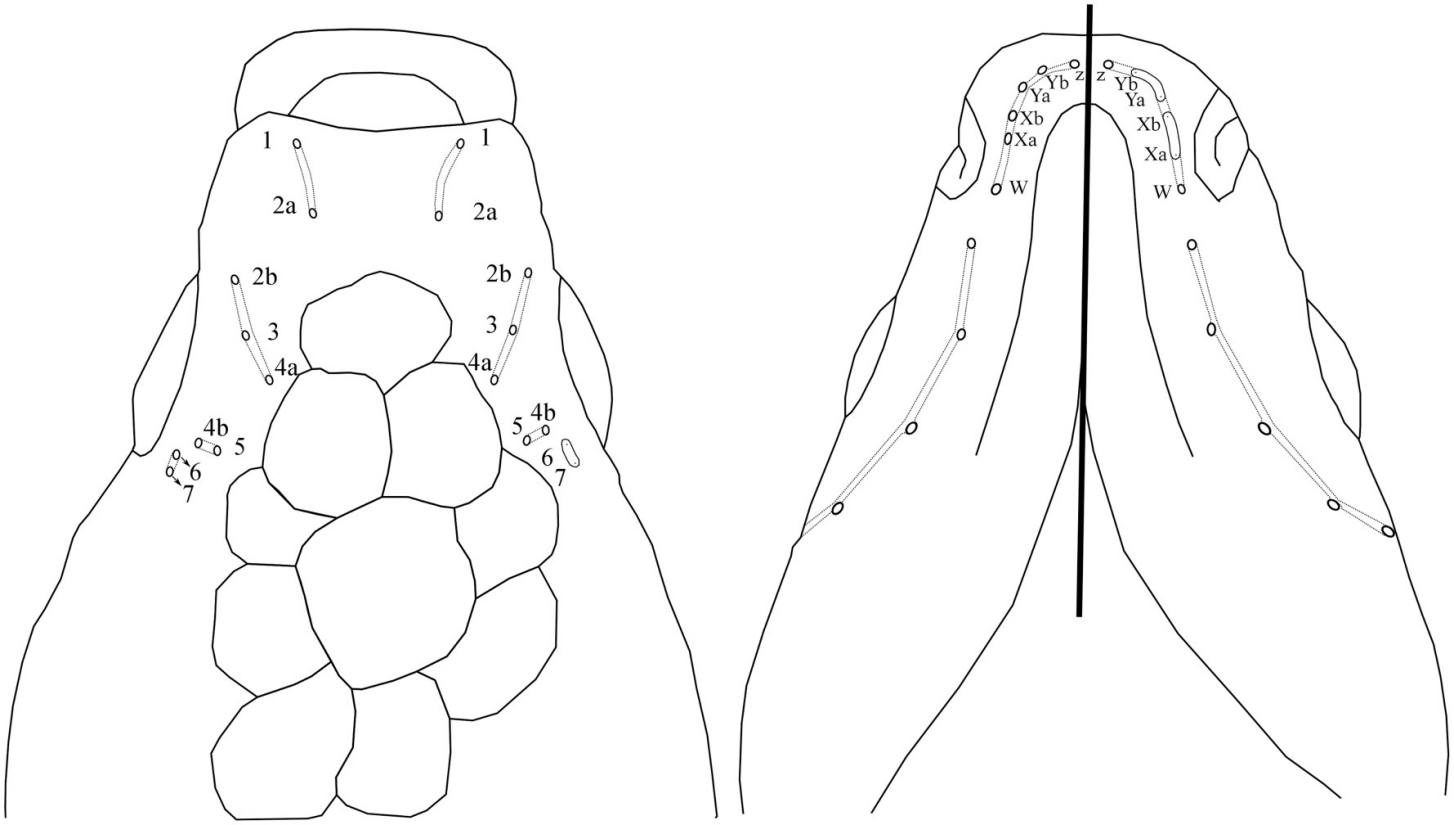
Schematic representation of head squamation and of the head lateral-line sensory system of *Jenynsia sulfurica*.

*Jenynsia sulfurica* is distinguished from *J*. *alternimaculata* by the presence of pore “W” (Fig 4) on the mandibular canal (vs. absence) and the higher number of gill rakers on the ventral arm of the first gill arch (14 vs. 9–12); from *J*. *onca* (Lucinda, Reis & Quevedo) by the lack of a dorsal convex expansion at subdistal segments of the right half of the sixth anal-fin ray of adult males (vs. presence); from *J*. *sanctaecatarinae* (Ghedotti & Weitzman) by the absence of a distinct rounded spot on the dorsal pectoral-fin base (vs. presence); from *J*. *obscura* (Weyenbergh) by the lower number of predorsal scales (13–16 vs 19–25); from *J*. *luxata* (Aguilera, Mirande, Calviño & Lobo) by the medial processes of left and right pelvic bones that developed and overlap each other at ventral midline (vs. processes reduced and not overlapping); and from *J*. *lineata* and *J*. *darwini* by the absence of a swelling between the urogenital opening and anal-fin origin. Additionally, *J*. *sulfurica* possesses a longer head (30.5–34.7% SL in males and 30.3–33.6% SL in females) than *J*. *luxata* (25.4–29.4% SL and 26.9–29.3% SL), *J*. *tucumana* (25.2–30.0% SL and 24.4–28.7% SL), *J*. *onca* (23.5–29.1% SL and 22.0–26.9% SL), *J*. *alternimaculata* (25.6–30.4% SL and 24.2–28.7% SL), and *J*. *sanctaecatarinae* (25.3–26.4% SL and 24.6–26.0% SL); and a longer postorbital (15.5–18.4% SL in males and 15.1–18.0% SL in females; and 49.3–54.1% of head length (HL) in males and 49.4–56.0% HL in females) than *J*. *tucumana* (11.7–13.5% SL and 11.4–14.1% SL), *J*. *onca* (37.0–43.0% HL and 36.4–47.3% HL), *J*. *alternimaculata* (11.0–15.1% SL and 10.7–14.0% SL), *J*. *sanctaecatarinae* (10.1–10.8% SL and 9.4–9.9% SL), and *J*. *lineata* (11.5–13.3% SL and 9.7–15.1% SL). Besides of the coloration pattern, *Jenynsia sulfurica* is distinguished from *J*. *maculata* by the contact of the lateral ehtmoid with the dorsolateral processes of vomer (vs. no contact), the intercalar small and restricted to point of the attachment of the lower limb of postemporal (vs. large intercalar), and the relatively short anal-fin ray nine along tubular gonopodium (vs. relatively long anal-fin ray nine).

### Description

Body stout, with a circular section in the anterior half and posterior half being laterally compressed. Head blunt; head squamation as in Fig 4. Mouth terminal, lower jaw slightly prognathous. Inferior lip hypertrophied, more noticeable in some specimens (Fig 2). Dorsal profile of body convex from snout tip to vertical line through anterior margin of eye, straight or slightly convex from this point to supraoccipital region, slightly convex to dorsal-fin origin, and slightly concave backwards to caudal-fin origin. Ventral profile of body straight from snout tip to vertical line through posterior eye margin, straight or slightly convex to anal-fin origin, and almost straight or slightly concave to caudal-fin origin. Maximum body depth at half-length between pectoral and pelvic fins. Sexual dimorphism present, males smaller than females and with intromittent organ formed by first eight anal-fin rays. Pectoral-fin distal tip reaching pelvic-fin insertion. Pelvic fin reaching gonopodial insertion in males, but never reaching anus in females. Dorsal-fin insertion at vertical line through center of anal-fin base in males, and at vertical line through or slightly anterior to anal-fin origin in females. Caudal-fin’s posterior margin straight or slightly convex. Absence of swelling between urogenital opening and anal-fin base of females.

Pores of cephalic sensory system associated with lateral sensory system (Fig 4) includes the supraorbital canal, with four branches, the first one containing pores 1 and 2a, second one includes pores 2b, 3, 4a, the third branch with pores 4b, 5 and the fourth branch includes pores 6 and 7. The pores of the last two supraorbital branches can be open or included in an open groove. Preopercular canal continuous, with 7 pores infraorbital canal formed by 4 pores; mandibular canal with pores Z, Ya and Yb separated or included in open groove, as well as pores Xa and Xb, and pore W, two or three rows of tricuspid teeth in both premaxilla and dentary.

Morphometric data in Table 1. Dorsal-fin rays 8* (27 specimens counted, * = indicates count of the holotype) or 9 (3). Anal-fin rays in females 10 (15). Principal caudal-fin rays 14 (1), 15 (12), or 16* (17). Pectoral-fin rays 16 (12), 17* (16), or 18 (2). Pelvic-fin rays 5 (1), 6* (29). Lateral line 30 (7), 31* (14), 32 (8). Predorsal scales 13 (2), 14* (11), 15 (13), or 16 (1). Circumpeduncular scales 16* (30). Vertebrae 30 (2), 31 (3), or 32 (1). Epipleural ribs 10 (5), or 11(1). Pleural ribs 11 (6). Gill rakers of first arch 14 (4).

**Table 1:** Morphometric measurements taken in *Jenynsia sulfurica* n. sp.; N = 30 individuals including the holotype; SD equals standard deviation.

	Holotype	Males			Females		
		Range	mean	SD	Range	mean	SD
Standard length	21.6	18.2–20.8	19.7	0.7	23.6–32.9	27.1	2.8
Percentage of SL							
Head length	30.7	30.5–34.7	32.2	1.0	30.3–33.6	31.8	1.0
Predorsal length	64.4	63.3–67.8	65.5	1.3	66.2–70.2	68.1	1.3
Snout to pectoral fin	33.0	32.7–36.7	34.3	1.2	32.7–35.8	34.4	1.0
Snout to pelvic fin	52.7	52.6–57.2	54.4	1.3	53.8–57.7	55.1	1.1
Peduncle depth	15.0	14.0–16.3	15.3	0.7	14.1–15.6	14.7	0.4
Caudal peduncle length	33.8	30.7–34.2	33.0	1.0	25.5–27.6	26.4	0.7
Gonopodium length	25.6	24.1–29.9	26.3	1.4	---	---	---
Percentage of HL							
Snout length	29.1	23.9–26.6	25.7	0.8	25.4–29.5	27.4	1.4
Post orbital length	51.4	49.3–54.1	51.2	1.7	49.4–56.0	53.2	2.2
Eye diameter	27.1	24.6–29.6	27.4	1.3	23.6–27.7	25.3	1.3
Interorbital width	40.1	37.3–44.0	40.9	1.5	40.5–47.8	43.5	1.8

### Color in life

Light silver to slightly golden body (Fig 2), with whitish belly and gular region with high guanine concentration. Ventral portion of the caudal peduncle light brown. Translucent opercle, reddish violet colored by the gills that are underneath. Golden to silver iris and black pupil. Scattered melanophores and some iridophores on margins of scales of dorsal region, forming a diffuse reticulate pattern, more conspicuous on the trunk. Concentration of melanophores in the middle portion of trunk and tail, forming diffuse, dark-grey vertical bands. Fins hyaline. Distal portion of gonopodium whitish. Reproductive females present two diffuse, red oval blotches, anterior to urogenital papilla, separated medially by a whitish area of guanine concentration. Pinkish pectoral girdle area when view ventrally due to superficial vascular irrigation. Dorsal region of head with scattered melanophores. Trunk dorsal portion with scattered melanophores on distal margin of scales. Golden thin longitudinal band anterior to dorsal-fin origin, about two scales broad. Medial portion of dorsal region of trunk and tail with uniformly scattered melanophores.

### Color after fixation

Body background pale yellow, darker at dorsal profile and lighter ventrally. Head dorsum, from snout tip to line through anterior margin of eye, with scattered dark-brown chromatophores, more densely concentrated on supraoccipital region. A diffuse mid-dorsal line of dark-brown chromatophores, from supraoccipital region to caudal-fin origin. Scales in this area with chromatophores bordering its posterior margin in a half-moon disposition. This pattern repeated in antero-dorsal half of body. Lachrymal area with concentrated chromatophores almost reaching distal-tip of maxilla, tapering as a line bordering the ventral and posterior eye-margins. Concentration of dark-brown chromatophores on upper margin of opercle forming a diffuse horizontal band, more apparent in some specimens. Eight to eleven irregular dashes along midlateral region of the body formed by dark-brown chromatophores, ranging from rounded spots to vertical bars, and occupying up to three scales (Fig 1). Gular region, between the mandibular canals of the cephalic sensory system, with few scattered dark-brown chromatophores. Some specimens with dark chromatophores concentrated on dorsal portion of pectoral-fin base. All fins hyaline with scattered chromatophores bordering the rays.

### Distribution

**Fig 5:**
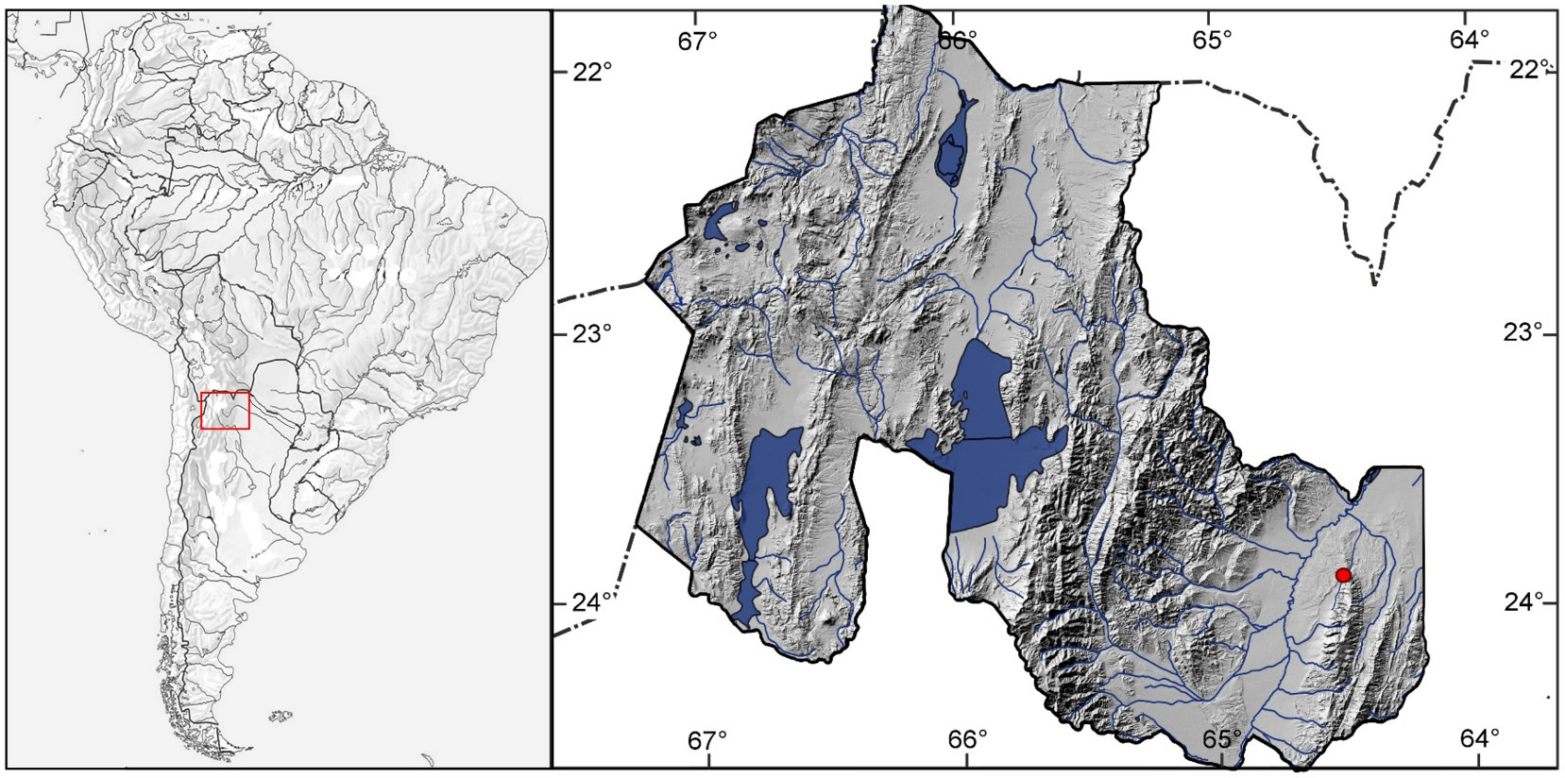
Hydrological map of South America. Red rectangle highlights the Province of Jujuy, where the type locality of *Jenynsia sulfurica* is indicated by a red dot. (Digital map from catalog.data.gov, U.S. Government Works subject to no copyright).

*Jenynsia sulfurica* is currently only known from Laguna La Quinta, which is part of a thermal system in the western flank of the Santa Bárbara hills, Santa Bárbara Department, Jujuy Province, Northwestern Argentina (Fig 5). It connects to the San Francisco River, tributary of the Bermejo River in the Paraná River basin.

### Ecological and behavioral notes

*Jenynsia sulfurica* inhabits an extreme environment, with elevated temperatures (39°-50°C) and low dissolved oxygen concentrations (0.25–5 mg/L). In the surroundings of the natural La Quinta lagoon, the sulfur odor (i.e., rotten egg-like smell) suggests the emission of H_2_S gas [32]. All these factors point to the presence of H_2_S in water, but its concentration has not been directly measured. Elevated levels of SO_4_ were reported in the lagoon (990 to 1015 mg/l; [33]), which in combination with the low oxygen levels also suggest the presence of H_2_S in water. In fact, the man-made Santa Barbara sulfur mine opens at the eastern margin of the lagoon. The water of the lagoon presents ClNa-type fluids, with total dissolved solids reaching up to 14700 mg/L, and pH values between 6.5 and 7.6 [32].

**Fig 6:**
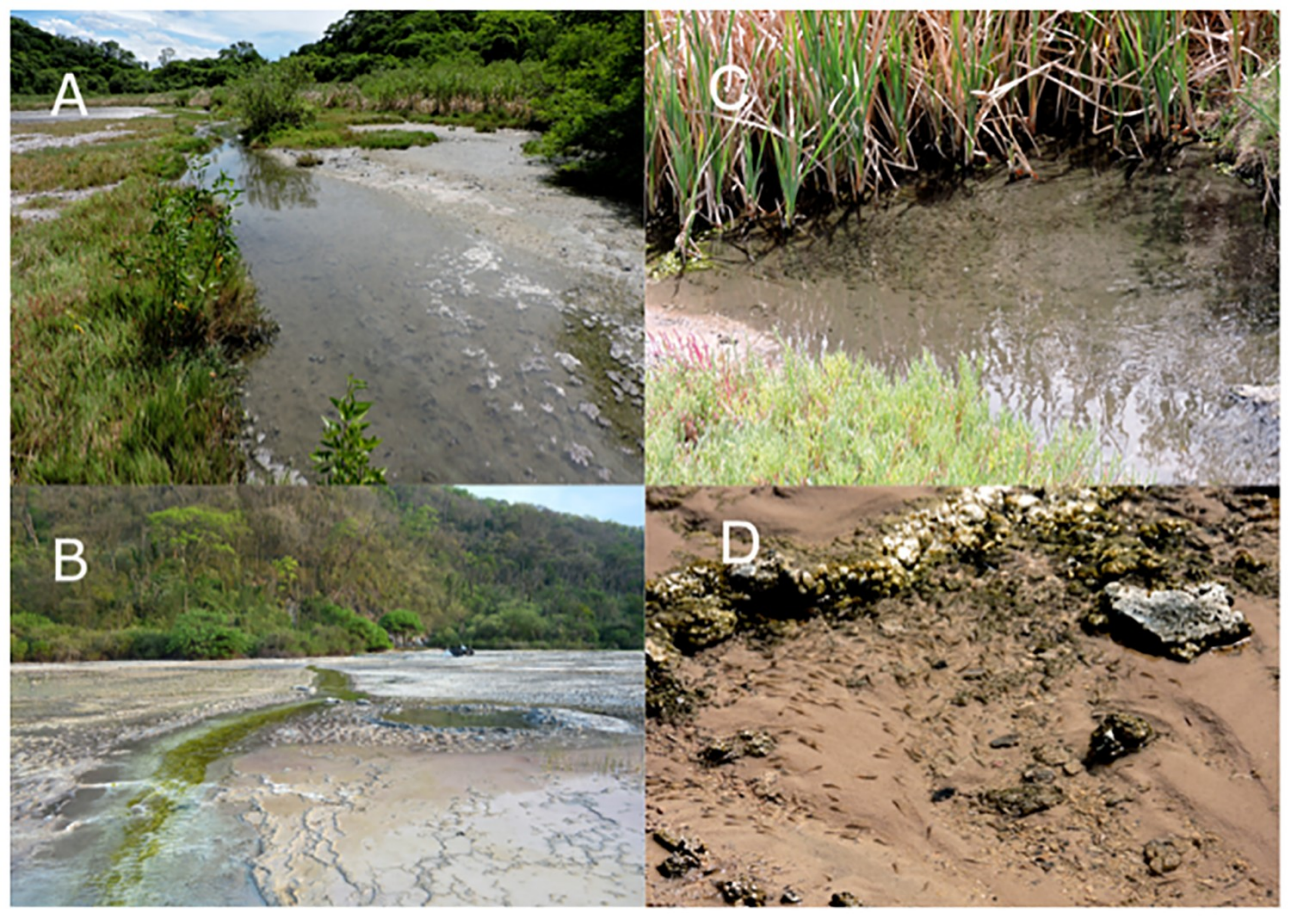
Photographs of the habitat of *Jenynsia sulfurica*. (A) the lagoon and (B) the sulfide spring which drains into the lagoon. (C) Pond of more than 10 cm water depth where adults of *J*. *sulfurica* were found and (D) small pond of 2 cm water depth inhabited by juveniles of *J*. *sulfurica*.

*Jenynsia sulfurica* is restricted to the lagoon and ponds located approximately 100 meters from the spring (Fig 6). Multiple attempts were made to localize this species in nearby environments, but these were all unsuccessful. Habitat segregation by age was observed. Adults were found mostly in the deepest ponds (i.e., of more than 10 cm in depth), which had low levels of dissolved oxygen (from 0.25 to 4.26 mg/l) and elevated water temperatures (40.8 to 42.3°C) (Fig 6C). Juveniles were found forming schools in small, shallow ponds (one or two cm deep), that had the highest levels of dissolved oxygen recorded in the area (5 mg/l) and a water temperature of 39.5°C (Fig 6D). Field and aquarium observations of this species show that it tends to form compact shoals that swim very close to the water surface.

### Etymology

The specific epithet “sulfurica” is a Spanish adjective, meaning “related to sulfur or from the sulfur”. In this case refers to the environment rich in sulfur that this species inhabits.

### *Jenynsia sulfurica* presents a single, unique *cox1* haplotype

**Fig 7:**
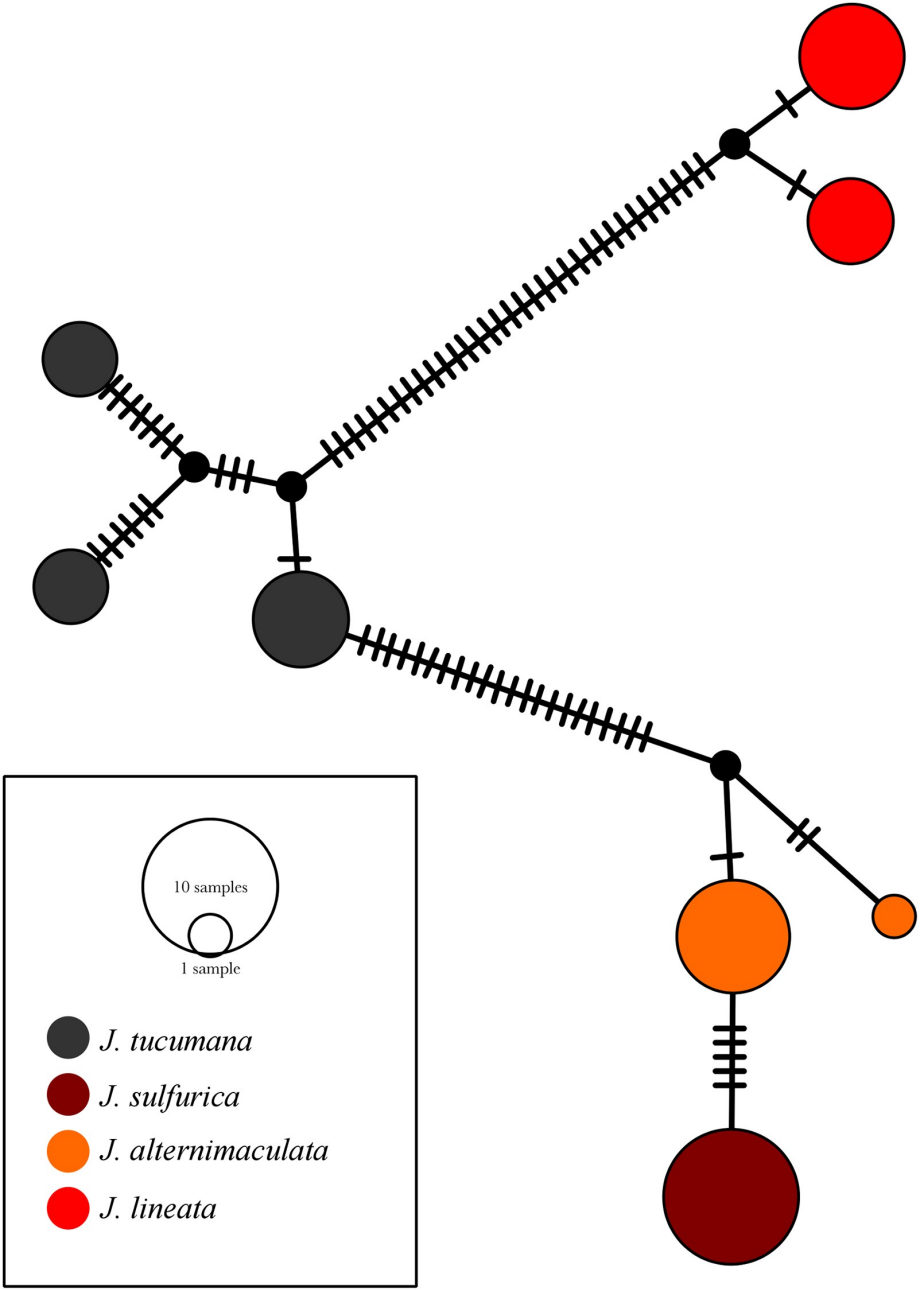
Haplotype network based on statistical parsimony of mitochondrial *cox1* gene.

A single and unique mitochondrial cox1 haplotype was found in the new species described here (Fig 7). This haplotype differs from those found in other species by a variable number of substitutions (Fig 7). It was most similar to the haplotypes found in specimens of *J*. *alternimaculata*. In this species, two haplotypes were found; the most common one differed by five point mutations from that found in *J*. *sulfurica* and the second one by eight point mutations. Of these mutations, two translate into amino acid substitutions (I75M and V128I, following *Poecilia sulphuraria* residue number). Three haplotypes were found for *J*. *tucumana* and two for *J*. *lineata*. These differed by over 20 point mutations from the group of haplotypes of *J*. *sulfurica* and *J*. *alternimaculata*.

### Phylogenetic relationships based on DNA, morphology and fossil taxa

**Fig 8:**
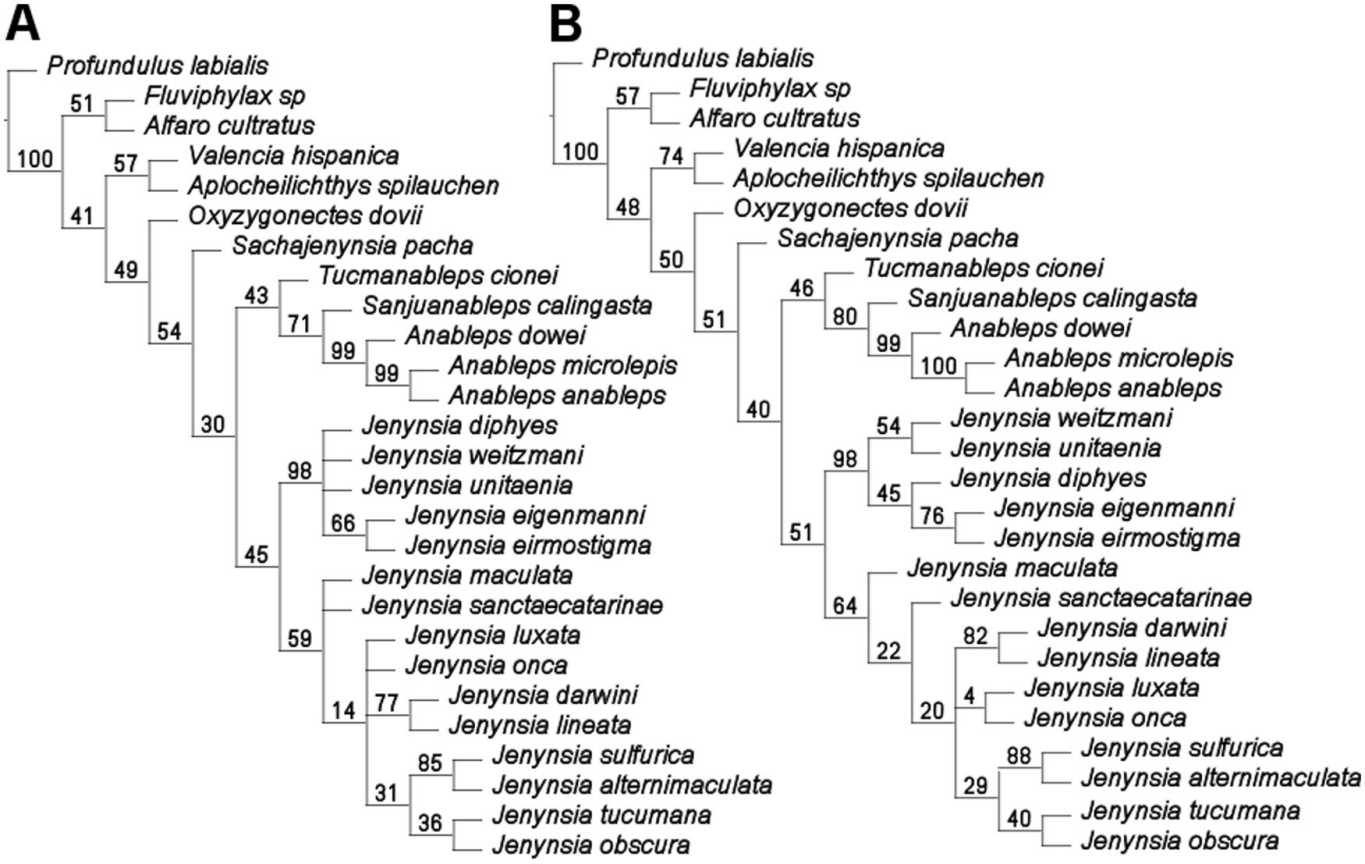
Topologies of most equally parsimonious trees and GC values. (A) equal weights and (B) implied weighting with supports measured under K = 8. Unsupported nodes are shown as collapsed. Families and outgroups are depicted for the first comprehensive analysis, combining morphological and molecular data.

Under equal weights, twelve most parsimonious trees of 1007 steps were found (CI = 57.2; RI = 59.3). The consensus tree topology under equal weighting was only partially resolved for both subgenera (Fig 8A). In the consensus tree, *J*. *sulfurica* was placed as sister taxa to *J*. *alternimaculata*, and this group as sister to a clade composed by *J*. *tucumana* + *J*. *obscura*. This clade formed a polytomy with *J*. *luxata*, *J*. *onca*, and a clade including *J*. *darwini* and *J*. *lineata*. In turn, this bigger clade formed a trichotomy with *J*. *maculata* and *J*. sanctaecatarinae. In the subgenus *Plesiojenynsia*, a politomy was observed including *J*. *diphyes*, *J*. *weitzmani*, *J*. *unitaenia* and a clade formed by *J*. *eigenmanni* + *J*. *eirmostigma*.

Under implied weighting, one most parsimonious tree of 1007 steps was found (CI = 57.2; RI = 59.3), in a wide range of concavities from K = 4 to K = 20. The tree topology (under K = 8; Fit: 42.51420) is totally resolved for the subgenera *Plesiojenynsia* and partially resolved for *Jenynsia* (Fig 8B). *Jenynsia sulfurica* was again recovered as sister taxa to *J*. *alternimaculata*, and this clade as sister of that composed of *J*. *tucumana* and *J*. *obscura*, as in the consensus tree under equal weights. This group formed a trichotomy with the groups *J*. *luxata* + *J*. *onca* and *J*. *darwini* + *J*. *lineata*. *Jenynsia maculata* was obtained as the sister group to all other species in the subgenus *Jenynsia*, with *J*. *sanctaecatarinae* branching next. The subgenus *Plesiojenynsia* was also completely resolved (Fig 8B).

Under equal and implied weights, *Sachajenynsia pacha* (Sferco, Herbst, Aguilera & Mirande) was basal to all anablepids, and *Tucmanableps cionei* (Sferco, Herbst, Aguilera & Mirande) and *Sanjuanableps calingasta* (Bogan, Contreras, Agnolin, Tomassini & Peralta) are successive sister taxa of *Anableps*.

## Discussion

### Phylogenetic relationships of *Jenynsia sulfurica*

The phylogenetic analysis herein performed under parsimony combining morphological and molecular data matrices and including known fossil taxa [34, 35], has resulted in a new hypothesis of relationships that present differences with previous hypothesis. Unlike the results presented by Bogan *et al*. [34], both under equal and implied weights, we recovered the monophyly of *Jenynsia*. In contrast to the last published phylogenetic analysis [36], the relationships of the subgenus *Jenynsia* were not totally resolved and the two clades within this subgenus were not recovered. Nevertheless, the phylogenetic placement of the three recently described extinct taxa agrees with that proposed by Sferco *et al*. [35], Bogan *et al*. [34], as well as the relationships of the subgenus *Plesiojenynsia* recently proposed by Amorim and Costa [36].

In particular, including *J*. *sulfurica* resolved the trichotomy in the subgenus *Jenynsia* seen in previous phylogenetic hypothesis including *J*. *tucumana*, *J*. *obscura* and *J*. *alternimaculata* [35]. The subgenus *Jenynsia* proposed by Ghedotti [25] was originally supported by three synapomorphies, the anal-fin ray 6 of males segmented on its proximal quarter (CH 48–1) and unsegmented on its distal quarter (CH 49–1), the vertical inclined proximal and middle radials associated with the first six anal-fin rays in adult males (CH 54–1). Later, Aguilera *et al*. [24] proposed four additional synapomorphies, the long posterodorsal process of palatine in dorsal view long (CH 17–1), the left and right hemitrichs of anal-fin ray six in adult males not laterally paired (CH 47–1), the absence of a protuberance on tip of tubular gonopodium formed by anal-fin ray eight (CH 51–1) and a series of three or more narrow lines not associated with distinct midlateral stripe on caudal peduncle present and continuous forming narrow lines (CH 64–1). The analysis herein performed has recovered the three synapomorphies proposed by Ghedotti [25] and the additional four later proposed by Aguilera *et al*. [24] as diagnostic for the subgenus *Jenynsia*, in contrast with Amorim [24] who recovered 6 from the 7 synapomorphies.

### Adaptations to sulfide springs: A case of convergence across fish families

*Jenynsia sulfurica* presents several morphological characters that appear to be adaptations to the presence of H_2_S in the La Quinta lagoon [32]. The enlarged head and opercular area are two of the most conspicuous characteristics of the new species that are unique among species of the genus *Jenynsia* (see Table 1 and ***Diagnosis*** above). Similar characteristics have been previously described for poeciliid fish inhabiting hydrogen sulfide-rich habitats [15, 18] and are proposed to be adaptations to the toxic conditions of these environments [10]. Enlarged head size allows for an increase in gill surface area, which is an advantage in oxygen poor environments in order to cover the high respiratory demands and the requirements of oxygen for sulfide detoxification [15]. Additionally, *J*. *sulfurica* was observed to swim close to the water surface and to have hypertrophic lower lips, both characteristics seen in other sulfide tolerant species and considered to be adaptive in oxygen-poor environments by facilitating access to the oxygen richer layer at interface between water and air [20, 37]. For example, poeciliid species from sulfide springs also spend a significant proportion of their time budget at the surface of the water [38], and often have evolved heritable or plastic morphological modifications of the lower jaw or the lower lip that maximize the uptake of oxygen from the water surface [18]. Finally, we found that *J*. *sulfurica*’s *cox1* gene differs from that of other *Jenynsia* species by having two non-synonymous substitutions that result in amino acid changes that are convergent with those observed in *Poecilia sulphuraria* and sulfide spring adapted populations of the *P*. *mexicana* species complex [21]. Of those, I75M has been suggested to provide a physiological mechanism related to living in sulfide-rich environments. The replacement of isoleucine by methionine at this residue results in a configurational change of the protein that affects the size of the D-pathway channel, still allowing for the passage of H_2_O but blocking H_2_S [21]. Together, these are remarkable examples of convergence across different fish families in their adaptation to the toxic conditions in freshwater sulfide springs, and contribute to the ongoing discussion about the predictability of evolution [39–41].

### Potential causes of reproductive isolation

Another species, *J*. *alternimaculata*, is found in nearby, non-sulfidic streams and rivers within the same drainage where *J*. *sulfurica* is found. This species presents a rather similar coloration pattern to *J*. *sulfurica* (i.e. elongate markings on the lateral surface of the body) and several osteological and genetic affinities that have resulted in the recovery of both species as sister taxa in the phylogenetic analysis. Nevertheless, the unique characteristic of *J sulfurica*, such as the absence of scales in the abdominal area and the different *cox1* haplotypes found in the two species, suggest that they have independent evolutionary histories. However, the mechanisms of reproductive isolation between these species are yet undetermined.

Divergent selection pressures can result in phenotypic differentiation of traits that confer a local fitness advantage when populations of a species occupy different environments [42]. In turn, such divergent natural selection could lead to the emergence of barriers to gene flow between nearby populations, resulting in ecological speciation [43]. The extreme abiotic conditions encountered in freshwater sulfide springs, such as the La Quinta lagoon which is inhabited by *J*. *sulfurica*, are known to impose different selection pressures to those experienced in nearby non-sulfidic habitats [5, 10, 17]. Additionally, natural and sexual selection against immigrants are known to contribute to the evolution of reproductive barriers between sulfidic and non-sulfidic populations of fish [38, 44]. Thus, the presence of H_2_S in the environment is recognized as an important driver of local adaptation and speciation [10]. Here, we present evidence suggesting that selection imposed by the presence of H_2_S has resulted in morphological and molecular divergence between *J*. *sulfurica* and its sister species, *J*. *alternimaculata*. It is possible that it has also contributed to the evolution of reproductive barriers between these species, and ongoing studies are aiming to determine the mechanisms involved in this process.

## Conclusions

So far, fishes of only four taxonomic families were known to have representatives in freshwater sulfide springs: Poeciliidae, Cyprinodontidae, Rivulidae (all Cyprinodontiformes), and Synbranchidae (Synbranchiformes) [10, 16]. Moreover, only six of these species are endemic to sulfide springs (i.e., *Cyprinodon bobmilleri*, *Aphanius ginaonis*, *Gambusia eurystoma*, *Limia sulphurophila*, *Poecilia sulphuraria*, and *P*. *thermalis*) [10]. *Jenynsia sulfurica* is the first species of the genus *Jenynsia*, and the only species within Anablepidae currently known to inhabit sulfide springs, a very extreme freshwater environment. Moreover, *Jenynsia sulfurica* is the first microendemism within the genus, being present only in a small sulfide thermal spring in Northwestern Argentina.

Members of the order Cyprinodontiformes are in general highly tolerant to high temperatures (e.g., [30, 45]), different concentrations of salinity (e.g., [31]), and a wide range of physicochemical environmental conditions (e.g., [29]), including environments with high concentrations of sulfide (e.g., [15]). In the genus *Jenynsia*, only a few studies were conducted to evaluate the tolerance of these fish to different environmental conditions, and almost all focused on *J*. *lineata* (= *J*. *multidentata*). This species is considered to be highly tolerant (e.g., euryhaline, eurythermic and euryoic), as it is commonly found to inhabit a wide range of abiotic conditions [26, 27, 46–50]. Although there are no studies in the remaining species of the genus, it is possible that closely related species also show high tolerance to variation in different abiotic factors. This is in line with the pattern of diversity within the genus, where different species are found across drainages (i.e., in the absence of gene flow) rather than within drainages (i.e., in the presence of gene flow). Thus, *Jenynsia sulfurica* is, from an ecological and an evolutionary perspective, an interesting species that attests to the extreme conditions of H_2_S enriched environments.

## Materials and methods

### Study area

We recently discovered a population of fish of the genus *Jenynsia* in a natural small, semicircular pond at the foothills of the Santa Barbara range, in “Ramal Jujeño”, Santa Barbara Department, Jujuy, Argentina, which is scented by sulfur odor and partially covered by brackish mud deposits [32]. This pond, named La Quinta lagoon, is fed by several internal and nearby hot springs that run through whitish riverbeds, indicative of mineral deposits (Fig 1). Both characteristics, the sulfur odor and mineral deposits, suggest the presence of H_2_S at high concentrations in the water [17]. Point measurements of dissolved oxygen and water temperature were taken at the collection site with a portable Oxymeter Hanna (HI9146).

### Specimens sampled and species description

Six specimens were cleared and counterstained following Taylor & Van Dyke [51]. Measurements are straight distances taken with a caliper to nearest 0.1 mm, following Aguilera & Mirande [52], and expressed as percent of standard length (SL; Table 1). Nomenclature of the sensory canal system of the head follows Gosline [53](Fig 3). For the description of the new species, the last two rays in the anal fin of females and dorsal fin of all specimens were counted as separate elements following Ghedotti & Weitzman [54]. The number of vertebrae includes the hypural complex as one element, and the gill rakers were counted from the ventral limb of the first gill arch. All measurements were compared to specimens of previously described species on the genus *Jenynsia* (S1 Appendix). All examined specimens were deposited at the ichthyological collection of the Fundación Miguel Lillo, Tucumán, Argentina (CI-FML) or the collection of the Instituto de Bio y Geociencias del Noroeste Argentino, Salta, Argentina (IBIGEO-I). All experimental procedures described were approved by the Animal Ethics Committee of the Fundación Miguel Lillo, Tucumán, Argentina, which consider animal welfare regulations. Collection permit was granted by the Ministerio de Ambiente de la Provincia de Jujuy (Permits 1103-306-M/2016).

### Phylogenetic analysis

To reconstruct a new phylogenetic hypothesis including the new species, we used a dataset that combined the morphological data published by Ghedotti [25] and subsequent additions to Bogan *et al*. [34], with molecular data of two markers (mitochondrial *cox1* and nuclear *sh3px3* genes) obtained from Genbank (accessions numbers in S2 Appendix), plus molecular data of the marker *cox1* sequenced and subsequently aligned in the course of this study. The analyzed matrix was composed of 1444 characters, including 1373 molecular (COX1 + SH3PX3) and 71 morphological characters. Phylogenetic analyses were performed using TNT software [55] under equal and implied weighting in a wide range of concavities (constant K) [56] using the protocol by Aguilera & Mirande [52] and Aguilera *et al*. [24]. Clade support was estimated using Symmetric Resampling, expressed as GC values (groups present/contradicted) [57]. The analyses were rooted in *Profundulus labialis* (Günther), and the outgroup includes *Fluviphylax obscurus* (Costa), *Aplocheilichthys spilauchen* (Duméril) and *Alfaro cultratus* (Regan). The analyses were performed both, with and without constraints in the outgroup structure as used by Ghedotti [25]. As previously done by Aguilera & Mirande [52] and Aguilera et al. [24], characters 19, 30, 40, 46, and 58 of Ghedotti’s [25] matrix were considered as additive (the list of character descriptions and possible character states used is included in the S1 Table).

### Sequencing of mitochondrial *cox1* gene

The mitochondrial *cox1* marker was PCR amplified using DreamTaq DNA Polymerase (Life Technologies, Carlsbad, USA). The size of the amplified PCR products was approximately 700 basepairs (bp; GenBank accession numbers: MN004782- MN004794), primers are listed in the Supporting Information. Primer annealing temperatures were 54°C (*J*. *lineata*, n = 10) or 58°C (*J*. *tucumana* (Aguilera & Mirande), n = 11, *J*. *alternimaculata* (Fowler), n = 8, *J*. *sulfurica n*.*sp*., n = 11). Purified templates were sequenced on an ABI 3130xl Genetic Analyzer (Life Technologies). The quality of sequencing reads was checked manually and reads were trimmed and assembled with SeqMan Pro (DNASTAR Lasergene). The trimmed DNA sequences used for the analyses constituted total lengths of 514 bp. We created a haplotype network estimation based on Statistical Parsimony (TCS, Templeton et al. 1992) as implemented in PopART version 1.7 (http://popart.otago.ac.nz).

### Nomenclatural acts

The electronic edition of this article conforms to the requirements of the amended International Code of Zoological Nomenclature, and hence the new names contained herein are available under that Code from the electronic edition of this article. This published work and the nomenclatural acts it contains have been registered in ZooBank, the online registration system for the ICZN. The ZooBank LSIDs (Life Science Identifiers) can be resolved and the associated information viewed through any standard web browser by appending the LSID to the prefix “http://zoobank.org/”. The LSID for this publication is: urn:lsid:zoobank.org:pub: 44A49BCD-237A-4277-820A-5926D2147536.

## Supporting information

S1 Appendix List of comparative material examined. (DOCX)

S2 Appendix GenBank accession numbers for genes used in the phylogenetic analysis and primers used to amplify and sequence *cox1*. (DOCX)

S1 Table Character states for *Jenynsia sulfurica* n. sp. A: polymorphic character, states 0 and 1. Description of character states are listed below the table.
(DOCX)

## References

[1] Amils R, Ellis-Evans C, Hinghofer-Szalkay HG. Life in extreme environments: Springer Science & Business Media; 2007.

[2] Riesch R, Tobler M, Plath M. Extremophile fishes Ecology, Evolution, and Physiology of Teleosts in Extreme Environments: Springer; 2015.

[3] Torres-Dowdall J, Karagic N, Plath M, Riesch R. Evolution in caves: selection from darkness causes spinal deformities in teleost fishes. Biology letters. 2018;14(6):20180197 10.1098/rsbl.2018.0197 29875208 PMC6030604

[4] Soares D, Niemiller ML. Sensory adaptations of fishes to subterranean environments. BioScience. 2013;63(4):274–83.

[5] Riesch R, Plath M, Schlupp I, Tobler M, Brian Langerhans R. Colonisation of toxic environments drives predictable life‐history evolution in livebearing fishes (Poeciliidae). Ecology letters. 2014;17(1):65–71. 10.1111/ele.12209 24188245

[6] Dassanayake M, Haas J, Bohnert H, Cheeseman J. Shedding light on an extremophile lifestyle through transcriptomics. New Phytologist. 2009;183(3):764–75. 10.1111/j.1469-8137.2009.02913.x 19549131

[7] Azua-Bustos A, González-Silva C, Arenas-Fajardo C, Vicuña R. Extreme environments as potential drivers of convergent evolution by exaptation: the Atacama Desert Coastal Range case. Frontiers in microbiology. 2012;3:426 10.3389/fmicb.2012.00426 23267354 PMC3526103

[8] Thomas D, Dieckmann G. Antarctic sea ice—a habitat for extremophiles. Science. 2002;295(5555):641–4. 10.1126/science.1063391 11809961

[9] McMullin ER, Bergquist DC, Fisher CR. Metazoans in extreme environments: adaptations of hydrothermal vent and hydrocarbon seep fauna. Gravitational and Space Research. 2007;13(2). 11543277

[10] Riesch R, Tobler M, Plath M. Hydrogen sulfide-toxic habitats In: Riesch R, Tobler M, Plath M, editors. Extremophile fishes Ecology, Evolution, and Physiology of Teleosts in Extreme Environments: Springer; 2015 p. 137–59.

[11] Kimura H. Hydrogen sulfide as a neuromodulator. Molecular neurobiology. 2002;26(1):13–9. 10.1385/MN:26:1:013 12392053

[12] Szabó C. Hydrogen sulphide and its therapeutic potential. Nature reviews Drug discovery. 2007;6(11):917 10.1038/nrd2425 17948022

[13] Bagarinao T. Sulfide as an environmental factor and toxicant: tolerance and adaptations in aquatic organisms. Aquatic Toxicology. 1992;24(1–2):21–62.

[14] Grieshaber MK, Völkel S. Animal adaptations for tolerance and exploitation of poisonous sulfide. Annual Review of Physiology. 1998;60(1):33–53. 10.1146/annurev.physiol.60.1.33 9558453

[15] Tobler M, Palacios M, Chapman LJ, Mitrofanov I, Bierbach D, Plath M, et al Evolution in extreme environments: replicated phenotypic differentiation in livebearing fish inhabiting sulfidic springs. Evolution: International Journal of Organic Evolution. 2011;65(8):2213–28. 21790570 10.1111/j.1558-5646.2011.01298.x

[16] Cochrane PV, Rossi GS, Tunnah L, Jonz MG, Wright PA. Hydrogen sulphide toxicity and the importance of amphibious behaviour in a mangrove fish inhabiting sulphide-rich habitats. Journal of Comparative Physiology B. 2019:1–13. 10.1007/s00360-019-01204-0 30719531

[17] Tobler M, Hastings L. Convergent patterns of body shape differentiation in four different clades of poeciliid fishes inhabiting sulfide springs. Evolutionary Biology. 2011;38(4):412–21.

[18] Palacios M, Arias-Rodriguez L, Plath M, Eifert C, Lerp H, Lamboj A, et al The rediscovery of a long described species reveals additional complexity in speciation patterns of poeciliid fishes in sulfide springs. PloS one. 2013;8(8):e71069 10.1371/journal.pone.0071069 23976979 PMC3745397

[19] Plath M, Tobler M, Riesch R, de León FJG, Giere O, Schlupp I. Survival in an extreme habitat: the roles of behaviour and energy limitation. Naturwissenschaften. 2007;94(12):991–6. 10.1007/s00114-007-0279-2 17639290

[20] Brauner CJ, Ballantyne CL, Randall D, Val A. Air breathing in the armoured catfish (*Hoplosternum littorale*) as an adaptation to hypoxic, acidic, and hydrogen sulphide rich waters. Can J Zool. 1995;73(4):739–44.

[21] Pfenninger M, Lerp H, Tobler M, Passow C, Kelley JL, Funke E, et al Parallel evolution of cox genes in H2S-tolerant fish as key adaptation to a toxic environment. Nature Commun. 2014;5:3873. 24815812 10.1038/ncomms4873

[22] Günther A. Catalog of the fishes of the British Museum VI. London, UK: Taylor and Francis; 1866.

[23] Amorim PF. *Jenynsia lineata* species complex, revision and new species description (Cyprinodontiformes: Anablepidae). J Fish Biol. 2018;92(5):1312–32. 10.1111/jfb.13587 29516517

[24] Aguilera G, Mirande JM, Calviño PA, Lobo LF. *Jenynsia luxata*, a new species from Northwestern Argentina, with additional observations of *J*. *maculata* Regan and phylogeny of the genus (Cyprinodontiformes: Anablepidae). Neotropical Ichthyology. 2013;11(3):565–72.

[25] Ghedotti MJ. Phylogeny and classification of the Anablepidae (Teleostei: Cyprinodontiformes) In: Malabarba LR, Reis RE, Vari RP, Lucena ZMS, Lucena CAS, editors. Phylogeny and classification of neotropical fishes. Porto Alegre, Brazil: Edipucrs; 1998 p. 561–82.

[26] Calviño P, Alonso F. First record of the genus *Jenynsia* from marine water on the coast of Punta del Este, Maldonado, Uruguay (Cyprinodontiformes: Anablepidae). J Fish Biol. 2016;88(3):1236–40. Epub 2016/01/29. 10.1111/jfb.12895 . 26817617

[27] Hued AC, de los Ángeles Bistoni M. Development and validation of a Biotic Index for evaluation of environmental quality in the central region of Argentina. Hydrobiologia. 2005;543(1):279–98.

[28] Karr J, Fausch K, Angermeier P, Yant P, Schlosser I. Assessing biological integrity in running waters: a method and its rationale. Illinois Natural History Survey, Champaign, Illinois, USA 1986;Special publication 5.

[29] Nordlie FG. Physicochemical environments and tolerances of cyprinodontoid fishes found in estuaries and salt marshes of eastern North America. Reviews in Fish Biology and Fisheries. 2006;16(1):51–106.

[30] Martínez JD, Cadena CD, Torres M. Critical thermal limits of *Poecilia caucana* (Steindachner, 1880)(Cyprinodontiformes: Poeciliidae). Neotropical Ichthyology. 2016;14(1).

[31] Ghedotti MJ, Davis MP. Phylogeny, classification, and evolution of salinity tolerance of the North American topminnows and killifishes, family Fundulidae (Teleostei: Cyprinodontiformes). Fieldiana Life and Earth Sciences. 2013;7:1–65.

[32] Miranda F, Johanis P. Geology and thermal features of El Ramal area, Jujuy province, Argentina. Proceedings of the World Geothermal Congress, Kyushu, Tohoku, Japan. 2000:1437–41.

[33] Pesce A, Miranda F. Catálogo de manifestaciones termales de la República Argentina. Vol I-II Región Noroeste SEGEMAR, Buenos Aires. 2003;36:1666–3462.

[34] Bogan S, Contreras VH, Agnolin F, Tomassini RL, Peralta S. New genus and species of Anablepidae (Teleostei, Cyprinodontiformes) from the Late Miocene of Argentina. Journal of South American Earth Sciences. 2018;88:374–84.

[35] Sferco E, Herbst R, Aguilera G, Mirande JM. The rise of internal fertilization in the Anablepidae (Teleostei, Cyprinodontiformes): two new genera and species from the Miocene of Tucumán, Argentina. Papers in Palaeontology. 2018;4(2):177–95.

[36] Amorim PF, Costa WJ. Reconstructing biogeographic temporal events in the evolution of the livebearer fish genus Jenynsia based on total evidence analysis (Cyprinodontiformes: Anablepidae). Systematics and Biodiversity. 2019:1–10.

[37] Chapman LJ. Low-oxygen lifestyles In: Riesch R, Tobler M, Plath M, editors. Extremophile fishes Ecology, Evolution, and Physiology of Teleosts in Extreme Environments: Springer; 2015 p. 9–33.

[38] Tobler M, Riesch R, Tobler C, Schulz‐Mirbach T, Plath M. Natural and sexual selection against immigrants maintains differentiation among micro‐allopatric populations. J Evol Biol. 2009;22(11):2298–304. 10.1111/j.1420-9101.2009.01844.x 19807829

[39] Orgogozo V. Replaying the tape of life in the twenty-first century. Interface Focus. 2015;5(6):20150057 Epub 2015/12/08. 10.1098/rsfs.2015.0057 . 26640652 PMC4633862

[40] Losos JB. Improbable destinies: Fate, chance, and the future of evolution: Riverhead Books New York, NY; 2017.

[41] Oke KB, Rolshausen G, LeBlond C, Hendry AP. How parallel is parallel evolution? A comparative analysis in fishes. The American Naturalist. 2017;190(1):1–16. Epub 2017/06/16. 10.1086/691989 . 28617637

[42] Kawecki TJ, Ebert D. Conceptual issues in local adaptation. Ecology letters. 2004;7(12):1225–41.

[43] Nosil P. Ecological speciation: Oxford University Press; 2012.

[44] Plath M, Pfenninger M, Lerp H, Riesch R, Eschenbrenner C, Slattery PA, et al Genetic differentiation and selection against migrants in evolutionarily replicated extreme environments. Evolution. 2013;67(9):2647–61. 10.1111/evo.12133 24033173

[45] Beitinger TL, Bennett WA, McCauley RW. Temperature tolerances of North American freshwater fishes exposed to dynamic changes in temperature. Env Biol Fish. 2000;58(3):237–75.

[46] Thormählen de Gil AL. Estudio biológico y experimental de las adaptaciones (eurihalinidad) del pez vivíparo *Jenynsia lineata*. Revista del Museo de La Plata. 1949;5.

[47] Menni RC, Gomez SE, Armengol FL. Subtle relationships: freshwater fishes and water chemistry in southern South America. Hydrobiologia. 1996;328(3):173–97.

[48] Butí C. El arroyo India Muerta y su ictiofauna (Tucumán, Argentina). Acta Zoológica Lilloana. 1998;44(2):309–12.

[49] Betito R. Comparação da complexidade das adaptações bioecológicas de dois peixes (*Jenynsia multidentata* e *Poecilia vivipara*)(Cyprinodontiformes) no estuário da Lagoa dos Patos (RS-Brasil). Revista Didática Sistêmica. 2006;3:71–100.

[50] Iglesias C, Mazzeo N, Goyenola G, Fosalba C, TEIXEIRA DE MELLO F, Garcia S, et al Field and experimental evidence of the effect of Jenynsia multidentata, a small omnivorous–planktivorous fish, on the size distribution of zooplankton in subtropical lakes. Freshwater Biology. 2008;53(9):1797–807.

[51] Taylor WR, Van Dyke GC. Revised procedures for staining and clearing small fishes and other vertebrates for bone and cartilage study. Cybium. 1985;9:107–19.

[52] Aguilera G, Mirande JM. A new species of Jenynsia (Cyprinodontiformes: Anablepidae) from northwestern Argentina and its phylogenetic relationships. Zootaxa. 2005;1096:29–39.

[53] Gosline WA. The sensory canals of the head in some cyprinodont fishes, with particular reference to the genus *Fundulus*. Occasional Papers of the Museum of Zoology, University of Michigan. 1949;519:1–17.

[54] Ghedotti MJ, Weitzman SH. Descriptions of two new species of *Jenynsia* (Cyprinodontiformes: Anablepidae) from southern Brazil. Copeia. 1995:939–46.

[55] Goloboff PA, Farris JS, Nixon KC. TNT, a free program for phylogenetic analysis. Cladistics. 2008;24(5):774–86.

[56] Goloboff PA. Estimating character weights during tree search. Cladistics. 1993;9(1):83–91. 10.1111/j.1096-0031.1993.tb00209.x 34929936

[57] Goloboff PA, Farris JS, Källersjö M, Oxelman B, Ramírez MJ, Szumik CA. Improvements to resampling measures of group support. Cladistics. 2003;19(4):324–32.

