# Supplementary material for: Molecular and morphological convergence to sulfide-tolerant fishes in a new species of Jenynsia (Cyprinodontiformes: Anablepidae), the first extremophile member of the family
Source: PLoS One. 2019 Jul 10;14(7):e0218810. doi: 10.1371/journal.pone.0218810 (PMC6619989; doi:10.1371/journal.pone.0218810)
Supplement: S1 Appendix — (DOCX) [file pone.0218810.s001.docx]

**Supplementary material**

*Aguilera et al. Molecular and morphological convergence to sulfide-tolerant fishes in a new species of Jenynsia (Cyprinodontiformes: Anablepidae), the first extremophile member of the family*

**S1 Appendix. List of comparative material examined**

**Comparative material** (when numbers in parentheses indicate the number of measured specimens): *Jenynsia alternimaculata*. **Argentina.** CI-FML 3825, 16 (16), 4 C&S, 20.8-43.7 mm SL, Salta, Orán, río Anta Muerta, tributary of río Blanco, río Bermejo basin. **Bolivia.** CI-FML 3831, 41 (10), 22.2-37.8 mm SL, Tarija, unnamed river in Acheralitos, which flows to río Cambarí, río Tarija basin. *Jenynsia eirmostigma*. Brazil. CI-FML 7505, 2 (2), 30.5-34.4 mm SL. *Jenynsia lineata*. **Uruguay.** CI-FML 1440, 36 (10), 19.8- 43.2, Canelones, Canteras de Carrasco. CI-FML 5462, 26 (10), 1 C&S, 20.9-38.3 mm SL, Maldonado, laguna del Diario. **Argentina.** CIFML 1081, 3 (3), 23.0-23.8 mm SL, Tucumán, Lules, arroyo Calimayo (2 km from Ruta Nacional 38). CI-FML 1569, 11 (5), 20.1-43.2 mm SL, Córdoba, arroyo Las Mojarras, 2 km from lago San Roque. CIFML 3826, 15 (10), 2 C&S, 28.0-36.5 mm SL, Tucumán, Monteros, río Mandolo, río Salí basin. *Jenynsia luxata*. Argentina. CI-FML 5464, holotype, 24.6 mm SL, male, Argentina, Tucumán, Burruyacu city, a small unnamed stream, río Tajamar basin. CI-FML 5465, 6 (6), 2 C&S, 20.9-29.1 mm SL; and MACN-Ict 9769, 5 (5), 25.1-35.9 mm SL, same data as for holotype. CI-FML 5466, 12 (12), 3 C&S, 17.3-44.3 mm SL, Santiago del Estero, Pellegrini, small flooded pools at Ruta Provincial 4, not connected to but near to río Urueña basin. *Jenynsia maculata*. **Argentina.** CI-FML 3832, 10 (5), 21.0-30.2 mm SL, Catamarca, Fuerte Quemado, small tributary of río Santa María. CI-FML 4743, 20 (20), 2 C&S, 15.9-30.4 mm SL, Salta, río Calchaquí, Cachi. *Jenynsia obscura*. **Argentina.** CI-FML 2009, 288 (10), 21.8-54.7 mm SL, Catamarca, Hualfín, Los Nacimientos. CI-FML 5463, 10 (10), 2 C&S, 27.6-57.8 mm SL, Catamarca, río Vís Vís basin. *Jenynsia sanctaecatarinae*. **Brazil**. CI-FML 7507, 4 (4), 34.0-51.5, Santa Catarina, near Timbe do Sul. *Jenynsia tucumana*. **Argentina.** CI-FML 3828, holotype, 30.7 mm SL, Tucumán, Trancas, río Vípos, 5 km from Ruta Nacional 9. AI 163, paratypes, 6 (6), 26.3-40.4 mm SL; ANSP 180781, paratypes, 6 (6), 20.4-33.0 mm SL; CI-FML 3829, paratypes, 4 (4), 26.2-32.8 mm SL; CI-FML 3840, paratypes, 2 C&S, 28.3 and 36.0 mm SL; CI-FML 3841, paratypes, 4 C&S, 20.4-38.5 mm SL, same data as holotype. *Jenynsia unitaenia*. Brazil. CI-FML 7506, 10 (10), 33.9-52.5, Santa Catarina, near Serra Furada.
