# Supplementary material for: Molecular and morphological convergence to sulfide-tolerant fishes in a new species of Jenynsia (Cyprinodontiformes: Anablepidae), the first extremophile member of the family
Source: PLoS One. 2019 Jul 10;14(7):e0218810. doi: 10.1371/journal.pone.0218810 (PMC6619989; doi:10.1371/journal.pone.0218810)
Supplement: S2 Appendix — (DOCX) [file pone.0218810.s002.docx]

**Supplementary material**

*Aguilera et al. Molecular and morphological convergence to sulfide-tolerant fishes in a new species of Jenynsia (Cyprinodontiformes: Anablepidae), the first extremophile member of the family*

**S2 Appendix. GenBank accession numbers for genes used in the phylogenetic analysis and primers for sequencing cox1**

GenBank accession for [cytochrome oxidase subunit 1](https://www.ncbi.nlm.nih.gov/nuccore/HQ682637.1/)(COX1)

*Species Accession number Reference*

*Anableps anableps* LC154806 [1]

*Anableps microlepis* KY031536 [2]

*Jenynsia unitaenia* KY031537 [2]

*Jenynsia sanctaecatarinae* KY031538 [2]

*Jenynsia lineata* KY031542 [2]

*Jenynsia darwini* KY031566 [2]

*Jenynsia sp.nov. "sulfurica"* MN004782, MN004783 this study

*Jenynsia alternimaculata* MN004784, MN004785 this study

*Jenynsia tucumana* MN004786, MN004787 this study

MN004788, MN004789 , MN004790

*Jenynsia lineata* MN004791, MN004792 this study

*Jenynsia onca*  MN004793 this study

*Jenynsia maculata* MN004794 this study

*Oxyzygonectes dovii* MG937084 [3]

*Alfaro cultratus* LC153120 [1]

*Aplocheilichthys spilauchen* LC154816 [1]

*Profundulus labialis* HQ682637 [4]

* sequences have been submitted to Genebank (Submission ID:2227946) and GenBank accession numbers will be updated when obtained

GenBank accession for [H3 and PX domain-containing 3-like protein](https://www.ncbi.nlm.nih.gov/nuccore/KJ697462.1/)(sh3px3)
*Species Accession number Reference*

*Anableps anableps* KJ697462 [5]

*Anableps dowei* KJ697463 [5]

*Anableps* *microlepis* KY986651 [6]

*Jenynsia unitaenia* KY031617 [7]

*Oxyzygonectes dovii* KJ697515 [5]

*Jenynsia* *darwini* KY986655 [6]

*Jenynsia* *eigenmanni* KY986653 [6]

*Jenynsia eirmostigma* KY986652 [6]

*Jenynsia lineata* KJ697492 [5]

*Jenynsia* onca KY031617 [7]

*Jenynsia* sanctaecatarinae KY986656 [6]

*Jenynsia* weitzmani KY986654 [6]

*Profundulus labialis* KJ697557 [5]

*Aplocheilichthys spilauchen* KJ697467 [5]

*Alfaro cultratus* KJ697460 [5]

*Valencia hispanica* KJ697563 [5]

Primers used in this study.

Primer_cox1_F TGGGTCTACAATCCACCGCT

Primer_cox1_R GCTTGAAACCAGTTCATGGGG
