# Supplementary material for: Molecular and morphological convergence to sulfide-tolerant fishes in a new species of Jenynsia (Cyprinodontiformes: Anablepidae), the first extremophile member of the family
Source: PLoS One. 2019 Jul 10;14(7):e0218810. doi: 10.1371/journal.pone.0218810 (PMC6619989; doi:10.1371/journal.pone.0218810)
Supplement: S1 Table — A: polymorphic character, states 0 and 1. Description of character states are listed below the table. (DOCX) [file pone.0218810.s003.docx]

**Supplementary material**

*Aguilera et al. Molecular and morphological convergence to sulfide-tolerant fishes in a new species of Jenynsia (Cyprinodontiformes: Anablepidae), the first extremophile member of the family*

**S1 Table. Character states for *Jenynsia sulfurica* n. sp. A: polymorphic character, states 0 and 1. Description of character states are listed below the table.**

| 1 – 10 | 11 - 20 | 21 – 30 | 31 – 40 | 41 – 50 | 51 – 60 | 61 – 70 | 71 |
| --- | --- | --- | --- | --- | --- | --- | --- |
| 0000010000 | 1100001011 | 0101100000 | 0000001002 | 1011011110 | 1111010A00 | 0000000001 | 1 |

1 Nasals: 0. Large, broad and rounded posteriorly; 1. Small, narrow and forming a distinct point posteromedially

2. Anterior margin of frontals in adults: 0. Even of forming a notch medially; 1. Expanded anteriorly between nasals

3. Frontals dorsal to orbit: 0. Flat, not expanded dorsally above orbit to accommodate eyes; 1. Expanded dorsally above orbit to accommodate eyes

4. Posteriorly directed process on sphenotic: 0. Absent, lateral sphenotic narrow; 1. Present, lateral sphenotic broad

5. Epiotic processes in adults: 0. Short, not extending beyond first vertebra; 1. Long, extending beyond first vertebra; 2. Absent

6. Dorsolateral processes of vomer: 0. Do no contact lateral ethmoid; 1. Contact lateral ethmoid

7. Parasphenoid in region of orbit: 0. Narrow, with pronounced ventral keel; 1. Broad, with expanded lateral shelves and with a less pronounced ventral keel

8. Ascending processes of parashenoid in adults: 0. Contact prootic; 1. Do no contact prootic

9. Prootic bridge over lateral canal and trigeminofacialis chamber of prootic: 0. Narrow; 1. Broad

10. Intercalar: 0. Small, restricted to point of attachment of the lower limb of posttemporal; 1. Large, elongated, extending laterally beyond point of attachment of the lower limb of posttemporal; 2. Absent

11. Ossified process on ventrolateral marginof basioccipital at attachment of Baudelot’s ligament in adults: 0. Absent; 1. Present.

12. Teeth in outer and inner rows on dentary and premaxilla, at least in embryos and young individuals: 0. Unicuspid; 1. Tricuspid

13. Ascending process of premaxilla in adults: 0. Long and approximately triangular; 1. Absent or represented by a short pointed or rounded process

14. Dorsal process of maxilla: 0. Small, more narrow anteriorly than at its base; 1. Expanded medially more broad anteriorly than at its base

15. Dorsal process of maxilla: 0. Separated; 1. Meet along dorsal midline

16. Anterior, articulatory head of palatine in dorsal view: 0. Narrow; 1. Broad

17. Posterodorsal process of palatine in dorsal in dorsal view: 0. Short; 1. Long and somewhat narrow, expanded medially and narrow laterally

18. Posterodorsal process of alveolar arm of premaxilla: 0. Pointed; 1. Rouded

19. Anterior cleft in anguloartiualr: 0. Large, extending posteriorly beyond posterior border of Meckel’s cartilage; 1. Small, not extending posteriorly posteriorly beyond posterior border of Meckel’s cartilage; 2. Absent

20. Posterodorsal process of lachrymal bordering orbit: 0. Short process; 1. Long, narrow process; 2. Absent

21. Anterior margin of hyomandibula: 0. Straight or slightly concave; 1. Strongly convex, expanded anteriorly

22. Posteroventral process of hyomandibula: 0. Absent or indistinct; 1. Present and prominent, usually associated with rounding or abrupt change in shape of anterior shelf of preoperculum

23. Posteroventral process of hyomandibula: 0. Does not interdigitate with anterior shelf of preopercle; 1. Interdigitate with anterior shelf of preopercle

24. Dorsal process of subopercle: 0. Short, approximately as tall as the width at its base; 1. Long, taller than the width at its base

25. Number of branchiostegal rays: 0. Six; 1. Five

26. Basihyal shape: 0. Triangular, broad anteriorly with large cartilaginous cap; 1. Short and rectangular, narrow anteriorly with little cartilage

27. Teeth on second and third hypobranchials in adults: 0. Absent; 1. Present

28. Third hypobranchial size: 0 large, greater than one-half the length of the second hypobranchial; 1- small, less than one-half the length of the second hypobranchial

29. Teeth on third ceratobranchial in adults: 0. Absent; 1. Present

30. Teeth on fourth ceratobranchial: 0. Extending posteriorly beyond the anterior head of fourth ceratobranchial; 1. Restricted to anterior head of fourth ceratobranchial; 2. Absent

31. Number of vertebrae: 0. Less than 45; 1. Greater than or equal to 45

32. Number of caudal vertebrae: 0. Less than 26; 1. Greater than 26

33. Parapophyses of trunk vertebrae: 0. Moderate; 1. Enlarged, longer than length of centrum

34. Number of pectoral-fin rays: 0. Less than or equal to 19; 1 greater than 19

35. Posterior extension of dorsal enclousure of cleithrum: 0. Present with distinct ventral notch; 1. Present and straight or slightly curved ventrally; 2. Present with distinct anteroventrally curving hook; 3. Absent

36. Dorsal postcleithrum: 0. Present; 1. Absent

37. Ventral postcleithrum width: 0. Slender, similar in width to adjacent first pleural rib; 1. Broad, with a lamina flange that is wider than adjacent first pleural rib

38. Posterior process of pelvic bone: 0. Present; 1. Absent

39. Medial processes of left and right pelvic bones: 0. Overlapping; 1. Separate

40. Number of anal-fin rays: 0. 12 or greater; 1. 11; 2. 10; 3. 9; 4. 8

41. Male anal fin: 0. Almost identical to female anal fin; 1. Modified into fleshy tubular intromittent organ supported by more than three anal-fin rays; 2. Modified into rod-like intromittent organ supported by three anal-fin rays

42. Position of base of anal-fin ray one in adult male: 0. Symmetrical around the midline; 1. Offset to right or left of midline

43. Length of anal-fin ray two in adult males: 0. Relatively long, greater than one quarter of length of ray three; 1. Relatively short, less than one quarter of length of ray three

44. Anal-fin ray three in adult females: 0. branched; 1. Unbranched

45. Length of ray four in adult males: 0. Long, approximately as long as ray three; 1. Short, less than two thirds length of ray three

46. Length of anal-fin ray five in adult males: 0. long, approximately as long as ray three; 1. Intermediate, between one-quarter and three quarters length of ray three; 2. Short, less than one-quarter length of ray three

47. Left and right halves of anal-fin ray six in adult males: 0. Laterally paired and narrow; 1. Not laterally paired, with both sides expanded and visible laterally

48. Segmentation of proximal quarter of anal-fin ray six in adult males: 0. Absent; 1. Present

49. Segmentation of distal quarter of anal-fin ray six in adult males: 0. Present, usually segmented to distal tip; 1. Absent

50. Callosity on anal-fin ray eight in adult males: 0. Absent; 1. Present

51. Protuberance on tip of tubular gonopodium formed by anal-fin ray eight: 0. Present; 1. Absent

52. Length of anal-fin ray nine along tubular gonopodium: 0. Relatively long, extending to near or beyond region or urogenital opening; 1. Relatively short, extending only two-thirds to three-fourths length of gonopodium

53. Anteriormost proximal anal-fin radial in adult males: 0. Separated; 1. Fused to the subsequent proximal radial forming a structure composed of five or six fused proximal radials

54. Proximal and middle radials associated with first six anal-fin rays in adult males: 0. Anteriorly inclined, sixth proximal, middle and distal radials anteriorly inclined in oblique line; 1- Vertically inclined, sixth proximal, middle and distal radials arranged at angle with proximal and middle radials inclined vertically and distal radials inclined anteriorly.

55. Proximal anal radials associated with first seven anal-fin rays in adult males: 0. Inclined vertically or anteriorly but no forming gap between proximal radials seven and eight; 1. Inclined far enough anteriorly to form noticeable gap between proximal radials seven and eight

56. Seventh middle anal-fin radial in adult males and females: 0 similar in size to adjacent middle radials; 1. Enlarged because it is the sixth most anterior middle radial

57. Eighth distal anal-fin radial in adult males: 0. Similar in size to adjacent distal radials; 1. Enlarged with respect to adjacent distal radials

58. Dorsal-fin origin: 0. At vertical that pases approximately through center of anal-fin base; 1. At vertical that pases through or slightly anterior to anal-fin origin;2. At vertical that pases posterior to anal-fin base

59. Hypurals in adults: 0. Fused, forming a single continuous hypural plate; 1. Fused, forming two symmetrical dorsal and ventral hypurals elements.

60. Posteriormost mandibular cale pore in adults (Pore w): 0. Present, most posterior neuromast of mandibular canal enclosed and bounded by pores W and X (or Xa) in adults; 1. Absent, most posterior neuromast of mandibular canal reduced or absent and not enclosed in canal or recessed in trough

61. Anteriormost supraorbital pore in adults (pore 1): 0. Single; 1. Composed of two or three smaller pores

62. Midlateral stripe: 0. Absent; 1. Present.

63. Series of dorsolateral blotches: 0. Absent; 1. Present and composed of black or dark brown chromatophores

64. Series of three or more narrow lines not associated with distinct midlateral stripe on caudal peduncle: 0. Absent; 1. Present and continuous forming narrow lines; 2. Presente and composed of short, dash-shape markings

65. pupils in each eye divided into upper and lower portion by iris extensions and dark corneal band: 0. Absent; 1. Present

66. Anterior nares: 0. Nontubular; 1. Tubular

67. Sexual laterality in females: 0. Absent; 1. Present

68. Sexual laterality in males: 0. Absent; 1. Present

69. Swelling between urogenital opening and anterior anal-fin base: 0. Absent; 1. Present

70. Viviparity: 0 absent; 1. Present

71. Symmetry of anal-fin ray five in adult male: 0. symmetric; 1. asymmetric, with one side short and the other side intermediate.

References

1. Suzuki-Matsubara M, Murase Y, Moriyama A. Higashiyama Zoo DNA Barcode Project. unpublished. unpublished.

2. Amorim PF. *Jenynsia lineata* species complex, revision and new species description (Cyprinodontiformes: Anablepidae). J Fish Biol. 2018;92(5):1312-32.

3. Bermingham E, Reina RG, Sanjur O. Freshwater Fish of Panama. unpublished. unpublished.

4. Valencia-Díaz X, Espinosa-Pérez H. Comparative analysis of three species populations of Profundulus (Teleostei: Cyprinodontiformes) using two genetic markers. Mitochondrial DNA. 2011;22(1-2):19-21.

5. Pollux B, Meredith R, Springer M, Garland T, Reznick D. The evolution of the placenta drives a shift in sexual selection in livebearing fish. Nature. 2014;513(7517):233.

6. Amorim PF, Costa WJ. Multigene phylogeny supports diversification of four-eyed fishes and one-sided livebearers (Cyprinodontiformes: Anablepidae) related to major South American geological events. PloS one. 2018;13(6):e0199201.

7. Costa WJ, Amorim PF, Mattos JLO. Molecular phylogeny and timing of diversification in South American Cynolebiini seasonal killifishes. Mol Phylogenet Evol. 2017;116:61-8.
